# An Algorithm to Identify Compounded Non-Sterile Products that Can Be Formulated on a Commercial Scale or Imported to Promote Safer Medication Use in Children

**DOI:** 10.3390/pharmacy3040284

**Published:** 2015-11-11

**Authors:** Varsha Bhatt-Mehta, Robert B. MacArthur, Raimar Löbenberg, Jeffrey J. Cies, Ibolja Cernak, Richard H. Parrish

**Affiliations:** 1 Department of Clinical, Social and Administrative Sciences, College of Pharmacy and Department of Pediatrics, Medical School, University of Michigan, Ann Arbor, MI, 48109, USA; 2 Med4Kids Research Collaborative, Ltd., Edmonton, AB, T6M 2J9, Canada; E-Mails: info@meds4keds.ca; Jeffrey.Cies@tenethealth.com; ibicernak@yahoo.com; 3 Clinical Development, Pharmaceutics International, Inc., Hunt Valley, MD, 21031, USA; E-Mail: RMacarthur@pharm-int.com; 4 Pharmaceutical Sciences Division, Faculty of Pharmacy and Pharmaceutical Sciences, Edmonton, AB, T6G 2R3, Canada; E-Mail: Raimar@ualberta.ca; 5 Drug Discovery and Innovation Centre, University of Alberta, Edmonton, AB, T6G 2R3, Canada; 6 Drexel University College of Medicine, Philadelphia, PA, 19129, USA; 7 St. Christopher’s Hospital for Children, Philadelphia, PA, 19140, USA; 8 Military and Veterans’ Clinical Rehabilitation Research, University of Alberta, Edmonton, AB, T6G 2G4, Canada

**Keywords:** compounded medications, formulation development, patient safety, pediatric

## Abstract

The lack of commercially-available pediatric drug products and dosage forms is well-known. A group of clinicians and scientists with a common interest in pediatric drug development and medicines-use systems developed a practical framework for identifying a list of active pharmaceutical ingredients (APIs) with the greatest market potential for development to use in pediatric patients. Reliable and reproducible evidence-based drug formulations designed for use in pediatric patients are needed vitally, otherwise safe and consistent clinical practices and outcomes assessments will continue to be difficult to ascertain. Identification of a prioritized list of candidate APIs for oral formulation using the described algorithm provides a broader integrated clinical, scientific, regulatory, and market basis to allow for more reliable dosage forms and safer, effective medicines use in children of all ages. Group members derived a list of candidate API molecules by factoring in a number of pharmacotherapeutic, scientific, manufacturing, and regulatory variables into the selection algorithm that were absent in other rubrics. These additions will assist in identifying and categorizing prime API candidates suitable for oral formulation development. Moreover, the developed algorithm aids in prioritizing useful APIs with finished oral liquid dosage forms available from other countries with direct importation opportunities to North America and beyond.

## 1. Introduction

Childhood illnesses often require the use of medications under the premise of “off-label” use. In February 2014, the American Academy of Pediatrics (AAP) released a policy statement from its Committee on Drugs regarding the off-label use of drugs (medications) in children [1]. For the purpose of the statement the Committee defined “off-label” use as that use which is not included in the package insert (US Food and Drug Administration (US-FDA)-approved labeling) for that drug and is “neither experimentation nor research” [1]. Such off-label use of drugs in children does not require any oversight by any institutional committee, Investigational Review Board, or federal regulatory agency. Use of drugs without appropriate research in children can put this population at significant risk for adverse events. Off-label use combined with use of medication management systems designed for adults and adapted for use in children can compound the risk of adverse events due to increased potential for medication errors. We have addressed the issue of medication management systems elsewhere in this Special Edition [2]. Here, we make an attempt to identify other issues related to off-label use of drugs and present potential solutions. That is, an algorithm that identifies compounded non-sterile products (CNPs) suitable for commercial scale pediatric formulation development, or importation, to address the medical needs of children.

## 2. Why Is off-Label Use of Drugs in Children Still a Problem?

The issue of “off-label” use of drugs in neonates, infants and children has prevailed for many decades. Federal legislation to address this issue was first proposed in 1998 and reauthorized in 2012. Since that time, various government agencies, including US-FDA and the National Institute of Child Health and Human Development (NICHD), have worked together to develop product labeling for pediatric patients for existing and new drug molecules coming to the market, if the drug has been or could be used in children.

According to a recent study published in JAMA-Pediatrics, the legislation has produced 406 pediatric labeling changes; out of those only 24 include data on neonatal drug dosing [3]. Identified factors responsible for the lack of appropriate drug dosing data in children go beyond the efforts of the US-FDA and NICHD, which formed partnership almost two decades ago to address this issue and formed collaborations with academia and a number of other entities to enhance this knowledge. Current dosing regimens for most drugs used in neonates, infants and children are extrapolated from adult pharmacokinetic (PK)/pharmacodynamics (PD) data rather than PK and PD data derived for pediatric populations. A major barrier to generation of appropriate dosing regimens based on sound PK/PD data is lack of commercial availability of uniform and stable drug formulations. Many drug dosage forms used in pediatric populations today within health care institutions in North America and elsewhere are extemporaneously compounded and lack validated product uniformity. This results in significant variability in drug disposition (ADME) estimates when these products are studied in children, as it is often unclear whether measured ADME variation originates from the patient or the product. Thus, research in pediatrics is not only necessary for new drug moieties, but also for existing active pharmaceutical ingredients APIs. Variations in dose preparation can significantly affect bioavailability, plasma concentrations and therapeutic effect.

Effective delivery and viable manufacturing are essential to improve the clinical and commercial value of a pharmaceutical product. A reliable formulation will not only ensure optimal clinical outcomes, but also assure reproducibility in manufacturing and increased stability. This gap between commercial availability and clinical utility is bridged daily primarily by the efforts of compounding pharmacists, especially in the hospital setting, where APIs are extemporaneously compounded from adult solid oral dosage forms to provide medications that otherwise are not available to vulnerable pediatric populations, such as children with cardiovascular disease, cancer, cystic fibrosis, organ transplants, and other life threatening conditions.

It has been noted that these compounding practices, despite best efforts and intentions, do not incorporate the necessary manufacturing controls to assure reproducible product potency, stability, and purity. Formal efforts to assist pharmacists and other practitioners to compound the best products possible include methods and guidelines found in the United States Pharmacopeia, US-FDA regulations and guidance documents, professional working groups, and elsewhere [4]. Working groups focusing on the topic date back to at least 1977, when the American Society of Hospital Pharmacists Special Interest Group on Pediatric Pharmacy Practice started working on the issue. This group continues to meet to the current day [5].

When compared to Good Manufacturing Practices (GMPs), the manufacturing standards required by FDA for commercial products, there are multiple recognized deficiencies inherent in extemporaneous formulation-compounding suitable for pediatric patients, which include the following:

- Lack of specifications required for component development by compounding pharmacies.
- No onsite testing of active ingredients and excipients for purity, potency, content and stability.
- No onsite specifications or testing of product containers and closures.
- Site-to-site variations in compounding procedures, equipment, and the degree of product handling/manipulation.
- Lack of environmental control, which might lead to unintentional contamination and generation of degradation products due to inconsistent exposure to light, temperature and processing controls.
- Lack of testing of finished products for purity, potency, content or stability.
- Stability data for establishing expiry dates of compounded products are derived from published data, where preparation methods likely vary from local methods, or are simply default expiry periods defined by regional pharmacy regulations and “best practices”.
- Published preparation methods provide only a portion of the information needed to consistently prepare a stable potent final product.
- Limited options available to mask bad-tasting active ingredients.
- The dose administration technologies used such as droppers, syringes, scoops, spoons, *etc.*, vary between sites and between prescription fills.
- Weak regulatory oversight.

Unfortunately, there are a number of barriers to the development of pediatric drug formulations under GMPs, not the least of which are economic in nature [6]. The first barrier concerns the market size. The elderly population (65 years of age and older) spends, on average, about 10 times more than the pediatric population (0–17 years of age) on medications. With regard to production, development of a unique pediatric formulation can take up to two years or more, with a cost ranging from $8 million to $15 million [7]. The second barrier relates to the need for incorporating taste-masking methods suitable for children; whereas the third one is due to the need to develop dosage forms such as liquids, suspensions, small tablets, films and chewable tablets that can be easily administered to children.

In the following sections, we present an algorithm that incorporates clinical and market-oriented variables in relation to existing APIs commonly used in pediatric practice that lack mass-produced formulations suitable for younger patients. Further, we overlay an array of scientific factors that support the selection of these APIs for mass-production, including Biopharmaceutical Classification System (BCS) and hot-melt extrusion (HME) as a flexible production technique for low solubility APIs.

## 3. Development of the Algorithm for a Tiered Selection Model for Candidate APIs

### 3.1. Clinical and Market Considerations

**Figure 1:**
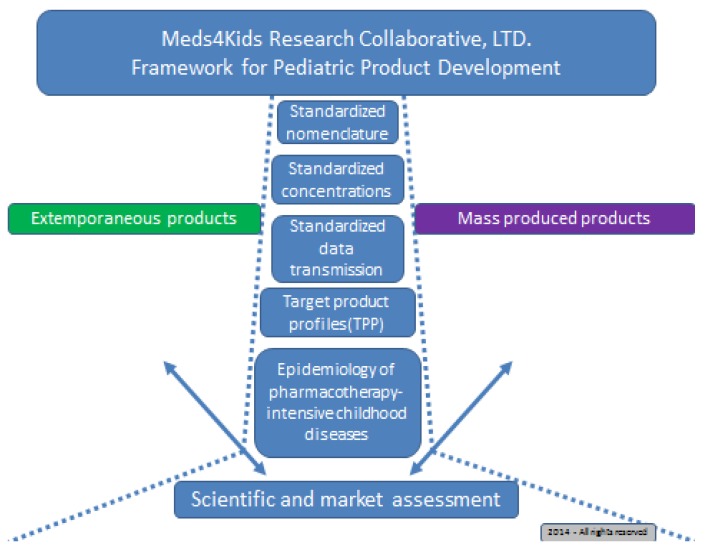
M4KRC framework for collaborative pediatric drug development.

Between September and December 2013, a North-American group of pharmacists, pediatricians, and pharmaceutical scientists formed Meds4Kids Research Collaborative, LTD (M4KRC). This group of clinicians and scientists with a common interest in pediatric drug development and medicines-use systems developed a framework for developing drugs in dosage forms suitable for use in pediatric patients (Figure 1). The group met in a series of six conference calls to create a list of candidate API molecules that possess the greatest clinical, scientific, and market potential for mass production. The proposed criteria for determining potential candidate API molecules were prioritized primarily based on Best Pharmaceuticals for Children Act (BPCA), which lists broad-based clinical developmental needs within pediatrics. Below are the first phase criteria that the group selected for the API molecules:

(1) Listed on BPCA priority document (clinical need—broad base);
(2) Available in an injection (API is stable in solution for at least 18–24 months);
(3) Not a federally controlled substance in Canada or the US (minimal paperwork for chain of custody);
(4) Potential applicability for oral adult market (for adult patients that cannot swallow tablets or capsules);
(5) Established indication for any age group in the official labeling of the API (immediate marketability through accelerated ANDA mechanism) and
(6) Off-patent in both Canada and the US, and available in a pharmaceutical grade powder from a reputable supplier.

The group reviewed the BPCA list and compared it to the list of 50 compounded non-sterile products (CNPs) prepared extemporaneously at St. Christopher’s Hospital for Children, all of which had scientific evidence for stability in a standardized concentration [8]. The clinicians (VBM, RHP, and JJC) adjudicated the list using a scoring system that assigned a score for each API molecule’s potential from a pharmacotherapy perspective. In the third meeting, a seventh factor was incorporated into the algorithm; the existence of a commercially-available oral liquid dosage form in the United Kingdom. Then, in the last two meetings, the scientists (RBM and RL) integrated the clinician-derived list with each API’s biopharmaceutical and mass-manufacturing properties that complemented and validated the clinical rankings.

The original list was then segmented into four tiers:

- The first tier included API molecules that met at least five of the six criteria;
- The second tier fulfilled four criteria;
- The third tier included API molecules not listed by name in the BPCA list but met all others except availability in the United Kingdom; and
- The fourth tier included those available in the UK that could be imported readily for pharmacokinetic (PK), pharmacodynamics (PD), and/or pharmacogenomics (PG) studies in children.

Several API molecules were removed from the list because of new market entry (enalapril), availability of one oral liquid strength (levetiracetam), known pharmaco-chemical problems (acetylsalicylic acid), or low use potential in children (pravastatin). The final, fourth tier API molecules identified based on criteria of potential viability as candidates ready for PK/PD/PG studies, are listed in Table 1. Of note, while sildenafil is available in a mass-produced product in the UK, the 10 mg/mL formulation too concentrated for use in younger infants children, thereby necessitating an extemporaneous 2.5 mg/mL strength. 

**Table 1:** M4KRC fourth tier candidate API molecules.

Generic Name	BCPA Listed?	Injection?	Non-Scheduled?	Oral Adult Market?	Established Adult Indication?	Off Patent?	Dose Form in UK?
Baclofen	1	1	1	1	1	1	Yes ^a^
Warfarin	1	1	1	1	1	1	Yes ^b^
Sildenafil	1	1	1	1	1	1	Yes ^c^
l-thyroxine	1	1-lyo	1	1	1	1	Yes ^d^

^a^ Available as 5 mg/mL oral solution; ^b^ Available as 1 mg/mL oral suspension; ^c^ Available as 10 mg/mL suspension powder; ^d^ Available as 5 microgram/mL and 20 microgram/mL oral solution

### 3.2. Scientific and Production Considerations—Applying BCS and HME

The next step for the group was to apply scientific principles of drug delivery and formulation techniques to the clinically-derived list that would enhance the likelihood of the commercial availability of a mass-produced product.

The Biopharmaceutical Drug Classification System (BCS) was introduced by Amidon *et al.* in 1995 [9]. Today, this classification system is accepted by most regulatory agencies like the US FDA, Health Canada and European Medicines Agency (EMA). APIs are classified into one of four quadrants according to their potential bioavailability [10]. Solubility and permeability are the two parameters used for the classification. Only if a drug can dissolve in the gastrointestinal tract can it be absorbed, and only if it is absorbed it can have a systemic effect. BCS class I drugs have high solubility and high permeability; class II are poorly soluble but highly permeable; class III are highly soluble but poorly permeable and class IV are poorly soluble and poorly permeable. Wu and Benet linked metabolism to the BCS classification, and showed that classes I and II drugs can be highly metabolized and be subject to transporters, while classes III and IV drugs are poorly metabolized or transported [11]. If two pharmaceutically equivalent drug products have similar dissolution behavior at pH 1.2, 4.5 and 6.8, then they can be considered therapeutically equivalent and bioequivalence is assumed without the need of an *in vivo* bioequivalence study [12].

To be able to adopt the BCS classification to paediatric drugs, some consideration have to be made: (a) what are the differences in drug metabolism and transport in the GI tract between adults and children in different age groups; (b) age-relevant changes in the physiology of the GI tract which can change the permeability or solubility classification (c) changes in permeability due to transporters in children. Such data could be developed in paediatric physiologic and population-based pharmacokinetic databases. The volume of fluid the drug is taken and paediatric dose strength will also be different in a paediatric BCS classification compared to the current BCS [13].

If these considerations are included, a BCS approach can be used in the development of pediatric dosage forms if a suitable strength exists. The knowledge of the BCS class helps pharmaceutical companies in drug development. For example, a class I drug might need much less formulation work compared to a class II drug where solubility must be improved [14]. The BCS classes of APIs with the highest potential for pediatric formulation development are listed in Table 2, and could be applied to all WHO-listed essential medicines for children [15,16,17,18]. One of the manufacturing methods that can address many of these barriers is the hot-melt extrusion (HME) technology [19,20,21]. HME disperses the active ingredient(s) as a matrix at the molecular level, thus forming solid solutions without the use of excessive heat or solvents while preserving drug potency. The HME approach has been successful in improving the delivery and human absorption of poorly water-soluble compounds. Extruded solid solutions (*i.e.*, the end product of HME) offer greater thermodynamic stability compared to products prepared by alternative processes such as spray drying, solvent evaporation and other hot melt methods.

**Table 2:** BCS Class and potential APIs for pediatric drug development.

API Generic Name	BCS Class	Reference
Metoprolol	II	[15]
Clopidogrel	II	[16]
Lisinopril	III	[16]
Amlodipine	I	[17]
Ursodiol	Uncl.	n.d.
Bosentan	Uncl.	n.d.
Pantoprazole	Uncl.	n.d.
Acetazolamide	IV	[17]
Spironoloactone	II/IV	[16]
Valaciclovir	I/III	[17]
Captopril	III	[18]
Nifedipine	II	[16]
Baclofen	Uncl.	n.d.
Warfarin	I	[16]
Sildenafil	I	[15]
l-thyroxine	III	[16]

Uncl. = unclassified; n.d. = no data.

Concerning economics, HME can be used to produce small (on a GMP scale) lots of a formulated product. The final production sizes of these small lot sizes, denoted by contract manufacturers as research and development batches or Clinical Trial Manufacturing (CTM) batches, are within the range of 10,000 to 50,000 units. The flexibility to produce small batches using HME process may provide a sufficient commercial market for a given pediatric population for a year or more. Compared to commercial tableting or capsule manufacturing approaches, which incorporate processes such as milling, mixing, spray drying, pre-compression, compression, tableting/encapsulation, HME is a much more economical process with reduced production time, fewer processing steps, and is suitable for continuous operation [21].

With regards to formulation attributes and taste masking, HME can produce final drug products with sustained, modified, and targeted release properties, and can also coat poor tasting products. So, the finished product can then be milled in a single step to form powders suitable for incorporation into child-friendly dosage forms such as mini-tablets, orally disintegrating tabs, and liquid suspensions. The output of the extrusion process, known as an extrudate, can be optimized to protect the API against changing pH conditions, light, heat, and moisture levels, thus confer good stability properties to the final formulation. While characterization of the pediatric gut awaits a more complete description, HME provides a useful tool for poorly soluble, highly permeable APIs [22,23,24].

An example of an API suitable for HME process is metoprolol succinate. Metoprolol is one of the most commonly used beta-blocking agents used in children from infancy through adolescence and into adulthood to treat a variety of cardiovascular conditions as an antihypertensive and heart rate modulator [25]. Metoprolol is a highly-soluble (~5 mg/mL) and permeable drug with no pH-dependent solubility. The required average pediatric dose ranges from 5 mg to 50 mg/day. Hence, a minimum concentration of about 2.5 mg/mL, with a maximum concentration of 10 mg/mL, is desired. When compounded in hospitals, a suspension with a concentration of 10 mg/mL is typically prepared. It is not possible to simply fill metoprolol succinate powder into a bottle to make a solution due to its poor solubility relative to dose volume and solution concentration as well as the formation of powder aggregates.

Metoprolol is an excellent candidate for HME as this process can increase in the dissolution rate of the drug for deriving clinically-appropriate concentrations, hence ease of reconstitution for a given strength. The process will also deliver good content uniformity of the drug and minimize risk for segregation. Overall, the manufacturing process is very simple, and involves only blending and processing using tight process controls. The HME process allows for in-process monitoring of the product quality using process analytical tools (e.g., Near Infra-Red (NIR) Spectroscopy). Moreover, there is no need for scaling up and down for different batch sizes.

In summary, the M4KRC-derived list of candidate API molecules has been derived from selection matrix that factored in a number of pharmacotherapy, scientific, production, and regulatory variables absent in other rubrics. These additions guided the group to identify the prime API candidates suitable for children in mass produced dosage forms. Further, the market-focused algorithm helped to prioritize useful APIs, and to facilitate clinical studies with finished oral liquid dosage forms available from other countries with direct importation potential to North America. Class II molecules are best suited for formulation using HME technologies Incorporating BCS classification and HME application into the final selection of candidate APIs, the following class II molecules are suggested for formulation and development: metoprolol, clopidogrel, spironolactone, and nifedipine. The assessment of these other variables, in addition to clinical and market parameters, is very important to increase the likelihood that a pharmaceutical manufacturer would successfully mass-produce and market these oral liquid dosage forms for children [26,27].

## 4. Conclusions

Successful public private partnerships have moved the science of pediatric clinical pharmacology forward. However, without reliable and reproducible evidence-based drug formulations designed for use in pediatric patients, safe and consistent clinical practices and outcomes assessments will continue to be difficult to ascertain. Identification and prioritization of prime candidate APIs with the algorithm has provided a broader integrated clinical, scientific, regulatory, and market basis, allowing for more reliable oral dosage forms for safe and effective medicines use in children of all ages.

The M4KRC group derived a list of candidate API molecules by factoring in a number of these pharmacotherapeutic, scientific, manufacturing, and regulatory variables into the selection algorithm that were absent in other rubrics. These additions have assisted in identifying and categorizing prime API candidates suitable for oral formulation development. Finally, the developed algorithm aids in prioritizing useful APIs with finished oral liquid dosage forms available from other countries with direct importation opportunities to North America and beyond. These finished and standardized oral dosage forms await further clinical testing.
